# Disease Surveillance on Complex Social Networks

**DOI:** 10.1371/journal.pcbi.1004928

**Published:** 2016-07-14

**Authors:** Jose L. Herrera, Ravi Srinivasan, John S. Brownstein, Alison P. Galvani, Lauren Ancel Meyers

**Affiliations:** 1 Department of Integrative Biology, The University of Texas at Austin, Austin, Texas, United States of America; 2 Departamento de Cálculo, Escuela Básica de Ingeniería, Facultad de Ingeneiría, Universidad de Los Andes, Mérida, Venezuela; 3 Applied Research Laboratories, The University of Texas at Austin, Austin, Texas, United States of America; 4 Department of Statistics and Data Sciences, The University of Texas at Austin, Austin, Texas, United States of America; 5 Department of Pediatrics, Harvard Medical School and Children’s Hospital Informatics Program, Boston Children’s Hospital, Boston, Massachusetts, United States of America; 6 Center for Infectious Disease Modeling and Analysis, Yale School of Public Health, New Haven, Connecticut, United States of America; Ecole Polytechnique Federale de Lausanne, SWITZERLAND

## Abstract

As infectious disease surveillance systems expand to include digital, crowd-sourced, and social network data, public health agencies are gaining unprecedented access to high-resolution data and have an opportunity to selectively monitor informative individuals. Contact networks, which are the webs of interaction through which diseases spread, determine whether and when individuals become infected, and thus who might serve as early and accurate surveillance sensors. Here, we evaluate three strategies for selecting sensors—sampling the most connected, random, and friends of random individuals—in three complex social networks—a simple scale-free network, an empirical Venezuelan college student network, and an empirical Montreal wireless hotspot usage network. Across five different surveillance goals—early and accurate detection of epidemic emergence and peak, and general situational awareness—we find that the optimal choice of sensors depends on the public health goal, the underlying network and the reproduction number of the disease (*R*_0_). For diseases with a low *R*_0_, the most connected individuals provide the earliest and most accurate information about both the onset and peak of an outbreak. However, identifying network hubs is often impractical, and they can be misleading if monitored for general situational awareness, if the underlying network has significant community structure, or if *R*_0_ is high or unknown. Taking a theoretical approach, we also derive the optimal surveillance system for early outbreak detection but find that real-world identification of such sensors would be nearly impossible. By contrast, the friends-of-random strategy offers a more practical and robust alternative. It can be readily implemented without prior knowledge of the network, and by identifying sensors with higher than average, but not the highest, epidemiological risk, it provides reasonably early and accurate information.

## Author Summary

As public health agencies strive to harness big data to improve outbreak surveillance, they face the challenge of extracting meaningful information that can be directly used to improve public health, without incurring additional costs. In this article, we address the question: *Which nodes in a social network should be selectively monitored to detect and monitor outbreaks as early and accurately as possible?* We derive best-case performance scenarios, and show that a practical strategy for data collection–recruiting friends of randomly selected individuals–is expected to perform reasonably well, in terms of the timing and reliability of the epidemiological information collected.

## Introduction

Public health agencies rely on diverse sources of information for detecting emerging outbreaks, situational awareness (e.g., estimating prevalence or severity), prediction of future burden, and triggering initiation of control measures. For influenza alone, the CDC has deployed at least eight different surveillance systems [1]. With the public health sector facing increasing budget constraints [2, 3], disease surveillance is at a critical juncture where next-generation big data can potentially be harnessed to revolutionize traditional data-limited practices and improve real-time situational awareness, early detection and forecasting of disease outbreaks.

HealthMap—an event-based system that aggregates worldwide news to generate global health risk maps—was among the first effective demonstrations of internet-driven surveillance [4, 5]. In 2009, Google Flu Trends—a detection algorithm for internet search queries of influenza-related terms—brought next-generation indicator-based syndromic surveillance to the forefront of public health [6–11]. It generally aligns well with seasonal dynamics in the US and Europe, but fell short during the 2009 H1N1 pandemic [12–14]. In the last few years, next-generation surveillance has exploded with efforts to combine both event and syndromic indicator data from search engines [15, 16], crowdsourcing (e.g., Flu Near You in the US and Influenzanet in Europe) [17, 18], Twitter (e.g., MappyHealth) [19, 20], and Facebook [21, 22]. While these new approaches are promising, public health agencies face the significant challenge of comprehensively integrating these diverse data sources to achieve specific surveillance objectives. Many next generation data sources, whether passively scraping data gathered for an incidental purpose or actively engaging volunteer participants, can be used to infer the underlying network through which disease, opinions or information spreads.

Decades of sociology and epidemiology research have demonstrated that network structure can profoundly influence the spread of disease and behavior, and determine if and when individuals are affected [23–30]. In particular, there are diverse methods for quantifying the importance or *centrality* of a *node* (individual) in a network, many of which have been shown to predict epidemiological risk and indicate optimal targets for interventions such as vaccination [31–37, 41–43].

In designing disease surveillance systems for networked populations, one seeks to identify nodes (*sensors*) that are likely to provide timely and accurate indications of epidemic activity. While analogous to the selection of efficient targets for vaccination on networked populations, the best sensors are not necessarily those most likely to be infected and infect others. Nodes that are the earliest or most often infected may be unreliable indicators of the broader epidemiological situation. Conversely, a representative cross-section of a network may provide accurate situational awareness, but the rate of detection from a representative cross-section may be too slow to serve as a timely trigger of control measures. Rapidity of targeted action during the initial phase of an outbreak is fundamental to the effectively curtailing transmission and minimizing disease burden. In previous work on livestock diseases, a network path based strategy has been proposed for identifying surveillance locations that would provide timely and accurate outbreak data [40]; in a recent analysis of disease surveillance in a high school population, Smieszek and Salathé introduce a promising sensor selection criteria (total time students spend collocated with other students) that is expected to yield timelier and more accurate information than alternative centrality-based criteria [47, 48]. Christakis et al. performed an experimental comparison of two social-network-based strategies in a college population [46]. In one strategy, the sensors were a random selection of students; in the other, the sensors were identified as friends of one or more random students. The *friends-of-random* surveillance group was expected to be biased towards more central individuals, and provided an indication of the 2009–2010 pandemic H1N1 influenza epidemic that was two weeks earlier than the *random* surveillance group.

Here, we use a mathematical model to systematically evaluate these and other strategies for selecting surveillance sensors across several networks and for an ensemble of common public health objectives. We quantify the timing and accuracy of the information gained by monitoring the disease states of strategically chosen sensors, as well as the robustness of the information across epidemiological scenarios characterized by different reproduction numbers, *R*_0_s. We find that the best surveillance targets are not always those with the highest epidemiological risk or those most representative of the underlying network.

## Methods

### Epidemic model

We simulate disease outbreaks in contact networks using a stochastic chain-binomial model that classifies the disease status of individuals as susceptible-exposed-infected-recovered (SEIR) [44, 45]. Networks consist of *nodes* representing individuals and *edges* between pairs of nodes representing contacts between individuals. The *degree* of a node is the number of other nodes to which it is connected via an edge.

During a simulated epidemic, each node is in one of four states: susceptible (S), exposed to disease but not yet infectious (E), infectious (I), or recovered (R). If a node *i* in state S shares an edge with a node *j* in state I, then *j* will infect *i* with probability *β* and *i* will transition from S to E. After a period of *l* days, *i* will enter the infectious state I. It will remain infectious for *d* days, and then move to the immune state R.

The *reproduction number* of a disease, denoted *R*_0_, indicates the growth rate of an epidemic and the expected number of secondary infections arising from a single infected host in an entirely susceptible population. Sustained epidemics are only possible when *R*_0_ > 1. In a random network, *R*_0_ is related to *β* as follows [49]:

$$\begin{array}{c} R_{0}=\beta \left(\frac{\left\langle k^{2}\right\rangle -\left\langle k\right\rangle }{\left\langle k\right\rangle }\right), \end{array}$$ (1)

where 〈*k*〉 and 〈*k*^2^〉 are the mean degree and the mean squared degree, respectively, of nodes in the network. *R*_0_ depends explicitly on both the intrinsic transmission rate of the pathogen and the structure of the network. For our analyses, we specify *R*_0_ and use Eq 1 to solve for the corresponding *β*. For the empirical networks considered, clustering, modularity and other non-random structures may cause the resulting *R*_0_ to differ slightly from the one initially specified.

For each simulation, we fix the latent period to *l* = 4 days and the infectious period to *d* = 7, roughly in the range of estimates for common respiratory diseases, including influenza [50, 51]. Epidemics are initialized with a single random infected node and allowed to evolve until there are no remaining infected nodes.

### Contact networks

Social interactions often generate complex network structures, with features that impose non-trivial constraints on the flow of information, behavior and disease [52–55]. We evaluated network-based surveillance strategies using three classes of social networks with distinct topological attributes.

1. *Scale-free networks*: Networks generated using the Barabasi-Albert algorithm [55] with *N* = 10,000 nodes, starting with *m*_0_ = 3 nodes and iteratively adding nodes with edges to *m* = 3 existing nodes.
2. *Student network*: A social network formed by *N* = 4,634 students (nodes) of the Engineering Department from Universidad de Los Andes in Merida—Venezuela, where edges indicate that students attended the same class during the fall 2008 semester (For more information refer to Supporting information S1 Fig).
3. *Montreal WiFi Network*: A co-location network for *N* = 103,425 users (nodes) of the Île Sans Fil free public wireless network in Montreal, Canada, where edges represent concurrent hotspot usage [56].

The degree distributions of the scale-free and Montreal network resemble power laws [55, 56], while the student network has a relatively homogeneous (Poisson) degree distribution. The Montreal network, but not the other two, exhibits strong community structure [56].

### Surveillance strategies

**Fig 1:**
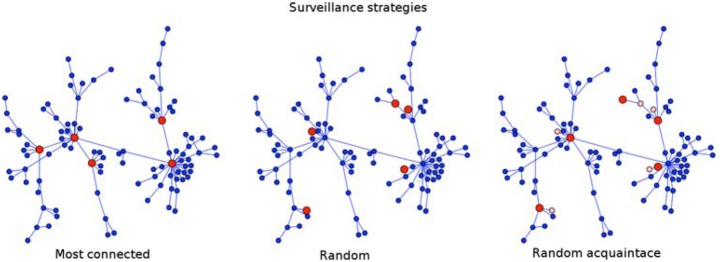
Schematic representation of the proposed surveillance strategies. Red circles indicate nodes that are selected to be surveillance sensors. For the random acquaintance strategy, yellow squares indicate randomly chosen nodes from which one random acquaintance was selected to be a surveillance sensor.

We propose three strategies for designing network-based surveillance systems. Each strategy is a criteria for selecting a subset of individuals to monitor for their disease state: (1) *most connected*: select the highest degree individuals in the network; (2) *random*: select individuals at random; and (3) *random acquaintance*: select a random acquaintance of random individuals (which should be biased toward high degree individuals [57]). These strategies are illustrated in Fig 1 for a scale-free network, where each surveillance subset includes five of the 100 nodes (in red).

The most connected strategy assumes complete knowledge of the network structure, whereas the random and random acquaintance strategies do not.

### Evaluation of surveillance strategies

**Fig 2:**
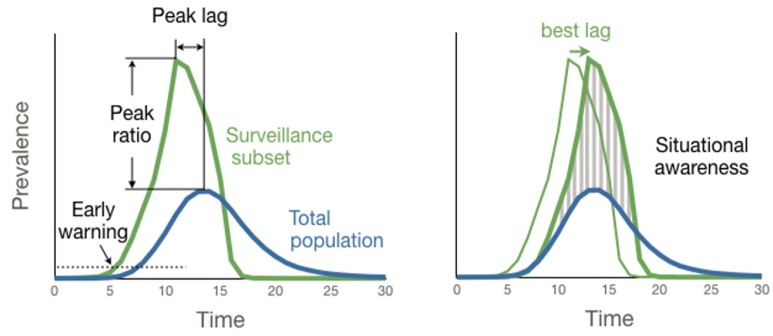
Surveillance objectives. To evaluate strategies, we compare the epidemic curve (prevalence time series) of the subset of nodes under surveillance (green lines) with the epidemic curve for the whole population (blue lines). We calculate the time lag between the surveillance group and whole population reaching 1% prevalence (*early warning*). We also calculate the time lag between the surveillance group and whole population reaching their epidemic peaks and the ratio of the magnitudes of the two peaks (*peak forecasting*), as well as the complement of the normalized mean absolute error (MAE)(*situational awareness*).

We assess the performance of each surveillance strategy with respect to four different public health goals, listed below (Fig 2). For each strategy-network combination, we build surveillance subsets by selecting 1% of all nodes (unless otherwise specified) via the strategy. We then estimate performance by running stochastic SEIR simulations, and make the following four comparisons between the prevalence time-series in the whole population to that of surveillance subset:

1. *Early warning*: The lag between the surveillance subset reaching 1% prevalence and the entire population reaching 1% [58–60] prevalence.
2. *Peak timing*: The lag between the surveillance subset reaching its epidemic peak and the entire population reaching its epidemic peak.
3. *Peak magnitude*: The ratio of peak prevalence in the surveillance subset and peak prevalence overall.
4. *Situational awareness*: The complement of the normalized mean absolute error (MAE), minimized over possible lags, is given by
  $$\begin{array}{c} 1-\min_{\lambda }\frac{\sum_{t}|\frac{x_{t}}{M}-\frac{y_{t+\lambda }}{N}|}{\sum_{t}\left(\frac{x_{t}}{M}+\frac{y_{t+\lambda }}{N}\right)}. \end{array}$$ (2)
  Here, *x*_*t*_ and *y*_*t*_ are the prevalence in the surveillance subset and in whole population at time *t*, respectively, *N* is the population size, *M* is the size of the surveillance subset, and λ is the lag.

All results are averaged over 2000 stochastic SEIR simulations. At the beginning of each simulation, the surveillance subset is chosen anew according to the given strategy. For each objective function, we quantify both the magnitude of the effect and its robustness with respect to a key epidemiological quantity, *R*_0_. High sensitivity of the information provided by a surveillance system to *R*_0_ indicates that the system may be unreliable or uninterpretable in situations where *R*_0_ is unknown or changing.

## Results and Discussion

**Fig 3:**
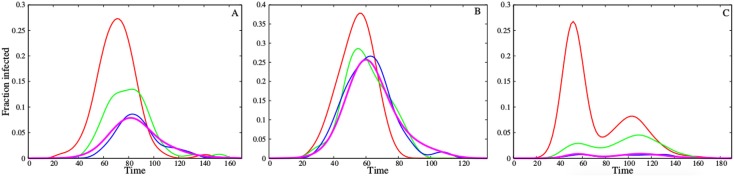
Typical epidemic curves for the three focal networks: (A) scale-free, (B) student and (C) Montreal. Lines indicate the fraction of infected nodes overall (magenta) and in 1% subsets of nodes selected according to the most connected (red), random (blue), and random acquaintance (green) surveillance strategies during a single SEIR simulation with *R*_0_ = 3.

In all three networks, the most connected strategy selects subsets of nodes that are most likely to experience earlier and more intense epidemics, whereas the random strategy yields collections of sensors that are highly representative of the population as a whole (Fig 3). The random acquaintance strategy produces subsets that provide some early warning in the scale-free and Montreal networks, but not in the highly homogeneous student network. The epidemic curves in the Montreal network occasionally exhibit multiple peaks, driven by underlying community structure [56].

**Fig 4:**
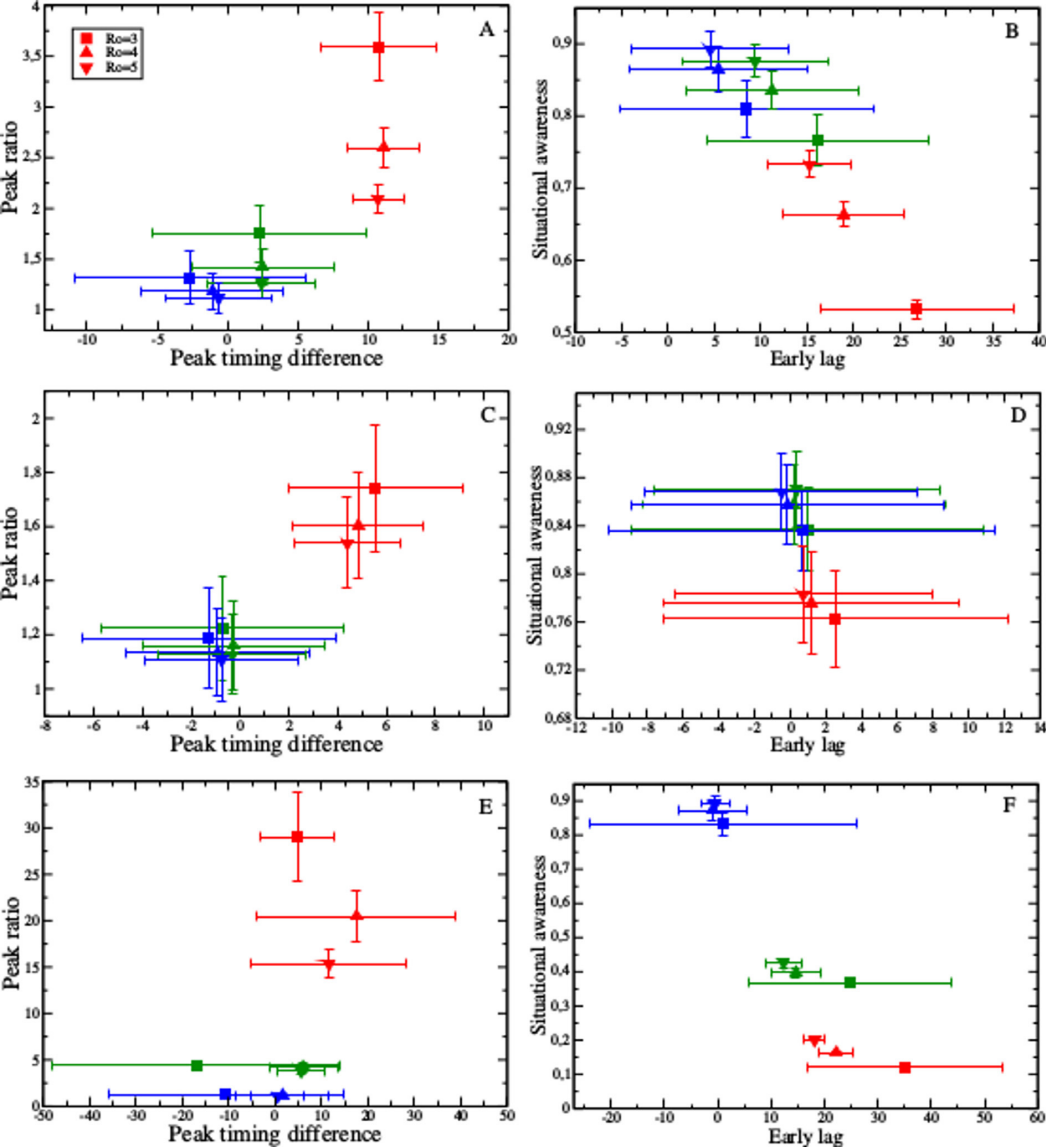
Performance of most connected (red), random (blue), and random acquaintance (green) strategies with respect to predicting the timing and magnitude of the peak (graphs A, C, and E), and achieving early warning and situational awareness (graphs B, D, and F). Points and error bars indicate mean and standard deviation in performance over 2000 simulations, respectively. Performance depends on both *R*_0_ and network structure: scale-free (graphs A and B), student (graphs C and D), and Montreal (graphs E and F).

A systematic evaluation of the three strategies in the three focal networks (Fig 4) shows that the most connected strategy consistently provides the earliest warning for both the beginning and peak of the season. The most connected strategy also exhibits the highest peaks and the least overall similarity to the full epidemic curve (Fig 4, red points). However, the timing of the early warning can be highly sensitive to *R*_0_, presenting a challenge when there is uncertainty regarding *R*_0_. For example, when *R*_0_ = 3, the most connected surveillance subset crosses the season onset threshold an average of 2.5, 27 and 35 days before the entire population in the student, scale-free and Montreal networks, respectively. When *R*_0_ = 5, these early warning periods decrease to averages of 0.71, 15,2 and 18.1 days, respectively (Fig 4B, 4D and 4F). The epidemic peak in the most connected surveillance subsets also depends on *R*_0_, reducing confidence in the estimation of peak burden under uncertainty (Fig 4A, 4C and 4E). In the Montreal network, the average ratio between the peak in the surveillance subset and the peak overall decreases from 29 to 15.3 as *R*_0_ increases from 3 to 5 (Fig 4E). In general, as *R*_0_ increases, the height of the epidemic peak in the entire population approaches that in the most connected subset of the population.

The random strategy yields surveillance systems that closely reflect the overall epidemiological dynamics, with early warning values close to zero and peak ratios close to one, across all networks and values of *R*_0_ (Fig 4, blue points). The random acquaintance surveillance groups perform relatively well in both the scale-free and Montreal networks (Fig 4A, 4B, 4E and 4F, green points). The random acquaintance approach offers helpful early warning for both season onset and peak, though not as much as the most connected group. Importantly, the random acquaintance approach exhibits greater robustness with respect to *R*_0_ in the timing of early warning, peak ratio, and situational awareness (overall correlation between surveillance epidemic curve and population epidemic curve) compared with the most connected group. However, in the student network, the random and random acquaintance subsets are virtually indistinguishable (Fig 4C and 4D). The student network is highly homogeneous, with most nodes having close to the average number of contacts. Thus, random acquaintances tend to be average as well.

**Fig 5:**
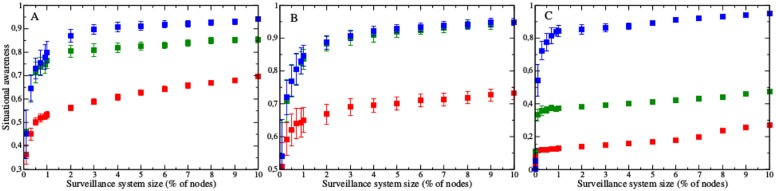
Size of surveillance systems impacts performance. Situational awareness (similarity between surveillance epidemic curve and full epidemic curve) improves as the surveillance system expands in the (A) scale-free, (B) student and (C) Montreal networks. Surveillance groups were chosen using the most connected (red), random (blue), and random acquaintance (green) strategies.

As the size of a surveillance system increases, the detected epidemic curves converge on the full epidemic curve, thereby improving situational awareness (Fig 5). In the scale-free and student networks, the performance of the three different surveillance strategies stabilizes to a quasi-stationary prevalence around 3%, which entails tracking 300 of 10,000 nodes and 139 of 4,634 nodes in the two networks, respectively. In the Montreal network, the random and random acquaintance groups reach their optimal performance by 0.5% (517 of 103,425 of nodes), while the most connected group is still improving beyond 10% (10,342 of 103,425 of nodes).

There are innumerable alternative strategies for selecting surveillance nodes, including prioritization based on other well-studied network centrality measures [38, 39]. For example, *k*-shell decomposition [37] and eigenvector centrality [53] are more computationally demanding and challenging to implement in practice, yet are not expected to significantly improve outcomes (S2 Fig).

### A theoretically optimal surveillance strategy

Following Newman [53], we use percolation theory to model SIR epidemics on networks, and derive the optimal surveillance group for early detection of an epidemic. We consider a disease with transmissibility *β* and recovery rate *γ* spreading through a network of size *N*. During the initial outbreak, the probabilities of each node being infected at time *t* are approximated by the vector

$$\begin{array}{c} x\left(t\right)=e^{(\beta \kappa -\gamma )t}v, \end{array}$$ (3)

where *κ* is the leading eigenvalue of the adjacency matrix and **v** its corresponding eigenvector [53].

We extend this equation to calculate the time lag between a subset *S* of the network of size *M* ≤ *N* reaching a given prevalence threshold *p* and the overall population prevalence reaching *p*. Let **1** be the vector of length *N* containing all ones, **1** = (1, …, 1), and **1**_*S*_ be the binary vector of dimension *N* indicating which *M* nodes are under surveillance

$$\begin{array}{c} 1_{S}=\left\{\begin{array}{cc} 1 & \text{if}\;\text{node}\;i\;\text{is}\;\text{in}\;\text{the}\;\text{surveillance}\;\text{subset}\;S\\ 0 & \text{otherwise.} \end{array}\right. \end{array}$$

For example, if the 1% most connected nodes were selected for surveillance in a network of size *N* = 1000, then the entries of **1**_*S*_ corresponding to the ten highest degree nodes would be one, and the remaining entries would be zero.

Let *τ* and *τ*_*S*_ be the times at which the entire population and a given surveillance group reach the prevalence threshold *p* and *p*_*S*_, respectively. Substituting into the above equation, we find

$$\begin{array}{c} p=\frac{x(\tau )\cdot 1}{N}=\frac{e^{(\beta \kappa -\gamma )\tau }v\cdot 1}{N} \end{array}$$ (4)

and

$$\begin{array}{c} p_{S}=\frac{x\left(\tau_{S}\right)\cdot 1_{S}}{M}=\frac{e^{\left(\beta \kappa -\gamma \right)\tau_{S}}v\cdot 1_{S}}{M}. \end{array}$$ (5)

To solve for the timing of early warning achieved through surveillance Δ*τ* = *τ*_*S*_ − *τ*, we equate *p* = *p*_*S*_,

$$\begin{array}{c} \frac{e^{(\beta \kappa -\gamma )\tau }v\cdot 1}{N}=\frac{e^{\left(\beta \kappa -\gamma \right)\tau_{S}}v\cdot 1_{S}}{M}. \end{array}$$ (6)

This implies

$$\begin{array}{c} \Delta \tau =\frac{1}{\beta \kappa -\gamma }\ln\left(\frac{c}{c_{S}}\right), \end{array}$$ (7)

where *c* = **v** · **1**/*N* and *c*_*S*_ = **v** · **1**_*S*_/*M* are the average eigenvector centralities in the network as a whole and the surveillance subset, respectively. The early season lag between the surveillance subset and the whole population can thus be positive or negative, and depends on ratio of their average eigenvector centralities.

**Fig 6:**
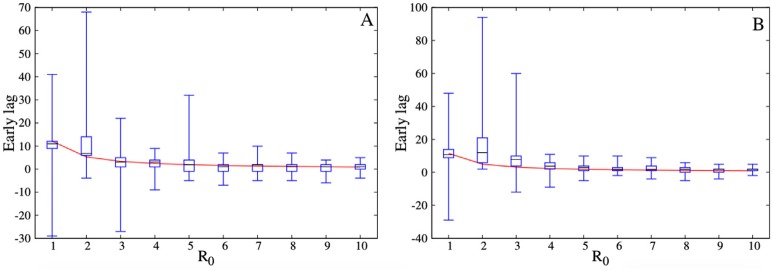
Comparing theory and simulation for early warning period in the scale free network. As *R*_0_ increases, the lag between the surveillance subset and the entire population reaching the early detection threshold decreases for both the (A) 1% highest eigenvector centrality nodes and (B) 1% highest degree nodes. Red curves indicate theoretical approximations; box plots show distribution of SIR simulation results.

We assessed the validity of this mean field approximation by comparing the expected early warning period (Eq 7) to simulated early warning periods for both the most connected subset and the subset of the 1% highest eigenvector centrality nodes. To match the assumptions of our mean field model, we simulated SIR rather than SEIR transmission dynamics. The simulations mirrored the theoretical expectations for both types of surveillance subsets in all three networks, as shown for the scale-free network (Fig 6).

Next, we solve for the surveillance subset that maximizes the length of the early warning period. For a given surveillance system size *M*, the earliest warning is achieved when **1**_*S*_ indicates the *M* nodes in the network with the highest valued entries in **v**. Thus, the theoretically optimal surveillance strategy for early warning of epidemic onset selects nodes with the highest eigenvector centrality.

Importantly, Δ*τ* depends on the disease parameters *β* and *γ*. Regardless of the choice of surveillance nodes **1**_*S*_, the timing of the early warning period will, therefore, increase as *R*_0_ decreases. An exception occurs when the average eigenvector centrality in the surveillance subset equals that in the population as a whole (*c* = *c*_*S*_). In that case, there is no early warning (Δ*τ* = 0). These properties are reflected in the sensitivity to *R*_0_ observed in our simulations (Fig 4B, 4D and 4F).

For the networks under consideration, the most connected strategy produces surveillance groups with relatively high eigenvector centrality while the random strategy yields groups with average eigenvector centrality. However, eigenvector centrality in random acquaintance groups depends on the underlying network: in homogeneous networks such as the student network, it will be average, whereas in heterogeneous networks, it will be above average.

### Identifying the optimal surveillance nodes

**Fig 7:**
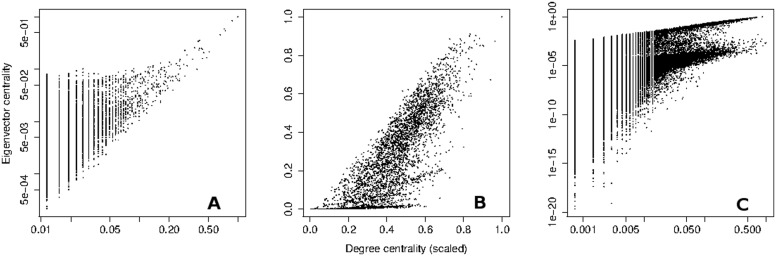
Scatter plots of eigenvector centrality vs. (scaled) degree centrality of nodes in the (A) scale-free, (B) student, and (C) Montreal networks. Both eigenvector and degree centralities are scaled to have maximum value 1, and log-log plots are shown for (A) and (C). The student network shows strong correlation between the two centrality measures, with a Spearman rank correlation coefficient of 0.819. For the scale-free and Montreal networks, the measures have more moderate rank correlation coefficients of 0.441 and 0.620, respectively.

Identifying individuals with the highest eigenvector centrality is challenging in real-world populations, where the underlying network structure is generally unknown. However, finding individuals with above average degree centrality is possible using local information. If eigenvector centrality is correlated to degree centrality, as it is in the three networks we consider (see Fig 7), it may be possible to use highly connected nodes as a proxy for high eigenvector centrality nodes.

One strategy for finding high degree centrality nodes is to follow chains of random acquaintances. This has been explored extensively in the context of respondent-driven sampling, such as chain-referral (i.e., “snowball”) sampling [64]. In particular, consider the simple random walk in which, at each step, the walker moves to a neighboring node selected uniformly at random. For connected, undirected networks, this is equivalent to the PageRank algorithm with no damping factor [53]. Assuming the network is fully connected, the distribution of the random walker after *m* steps approaches a stationary distribution as *m* → ∞, in which the probability of landing on a node is exactly proportional its degree [65]. Thus, the more connected the node, the more likely we are to reach it.

Precisely, let *k*_*i*_ be the degree of node *i* and *P*(*k*) the degree distribution of the network. The *n*th moment of the degree distribution is:

$$\begin{array}{c} \left\langle k^{n}\right\rangle =\frac{1}{N}\sum_{i}k_{i}^{n}=\sum_{k}k^{n}P\left(k\right). \end{array}$$ (8)

Let *D*_*m*_ denote the degree of the node at which the random walk resides on the *m*th step, starting from a node chosen uniformly at random. Assuming the mean degree 〈*k*〉 < ∞, then the distribution of *D*_∞_ is given by *kP*(*k*)/〈*k*〉. If 〈*k*^3^〉 < ∞, which is true for any finite graph but will be violated for power-law networks without cutoff, the mean and standard deviation of *D*_∞_ are given by

$$\begin{array}{c} \mu_{\infty }=\frac{\left\langle k^{2}\right\rangle }{\left\langle k\right\rangle },\;\sigma_{\infty }=\sqrt{\frac{\left\langle k^{3}\right\rangle }{\left\langle k\right\rangle }-{\left(\frac{\left\langle k^{2}\right\rangle }{\left\langle k\right\rangle }\right)}^{2}}. \end{array}$$ (9)

By comparison, the distribution of randomly sampled nodes (*D*_0_) has mean *μ*_0_ = 〈*k*〉 and standard distribution $\sigma_{0}=\sqrt{\langle k^{2}\rangle -{\langle k\rangle }^{2}}$. Thus, the random walk sample is biased towards nodes with larger degrees. For intermediate values of *m*, the distribution of *D*_*m*_ can only be derived with full knowledge of the underlying graph. Instead, this distribution converges to that of *D*_∞_ at a rate that depends on the second largest eigenvalue of the adjacency matrix of the graph. If this eigenvalue is close to one, which is usually the case for connected networks with high modularity, convergence is very slow and random walk sampling may require numerous steps to achieve its optimal performance. Methods that bias the random walk towards higher eigenvector centrality nodes should be more effective in this setting. For example, the maximal entropy random walk samples nodes proportional to eigenvector centrality just as the simple random walk considered earlier samples nodes according to their degree centralities [66]. However, the transition probabilities of the the maximal entropy random walk require global information about the network, making it impractical to implement without approximation as part of the sampling strategy.

**Fig 8:**
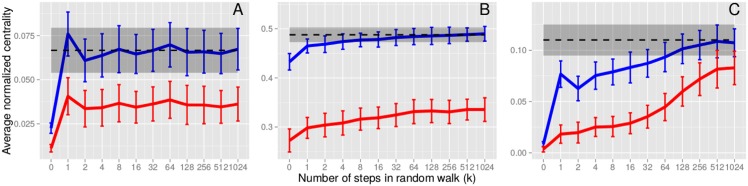
Random walks increase centrality in the surveillance subset. For purposes of comparison, degree (blue) and eigenvector centrality (red) are divided by the maximum degree and maximum eigenvector centrality, respectively, in each network. Mean degree approaches its theoretical limit (dashed lines), and mean eigenvector centrality also increases as the random walks progress in the (A) scale-free (subset contains *ϵ* = 1% of nodes), (B) student (*ϵ* = 2%), and (C) Montreal (*ϵ* = 0.1%) networks. As expected, the mean degree converges to a normal distribution with mean *μ*_∞_ and standard deviation *σ*_∞_ (gray shading) as the number of steps in the walk, *k*, increases. The random walks converge within a few steps in the scale-free and student networks, but require more steps in the highly modular Montreal network.

Eq 9 provide a theoretical upper bound to the mean centrality that can be achieved when using a random walk on a network to design a surveillance system. In particular, for a random-walk surveillance subset of size *M* = *ϵN* with fixed *ϵ* and large *N*, the empirical mean of the sample will become approximately normal with mean *μ*_∞_ and standard deviation $\sigma_{\infty }/\sqrt{M}$, as illustrated for our three study networks (Fig 8).

### Conclusions

The success of both traditional surveillance systems such as the U.S. Outpatient Influenza-like Illness Surveillance Network (ILINet) and next generation participatory systems including FluNearYou [17, 18], depends on targeted recruitment of reliable, informative providers. With Meaningful Use and the advent of digital disease detection, we are moving from an era of sparse, volunteer-based data into an era of data inundation [16, 61]. Nonetheless, we still face the challenge of finding reliable data sources. Effective mining of electronic medical records, social media and other internet source data, such as Google, Twitter or Facebook, requires sifting through petabytes of data for streams that can provide early and accurate information about emerging outbreaks. While random representative sampling is a good rule-of-thumb and has guided the development of numerous surveillance systems, we can improve the timeliness of surveillance by exploiting our evolving understanding of social networks and their impacts on infectious disease dynamics [24, 28–30, 45, 49, 52–55, 62, 63].

In an ideal scenario where both the contact network and the reproduction number (*R*_0_) of the disease are known in advance, public health agencies can monitor the most informative nodes and achieve very accurate and early assessments of emerging epidemics. For example, we find that surveillance of the most connected individuals in the Montreal WiFi network can increase lead time on detecting epidemic emergence by two to three weeks and anticipating the epidemic peak by over a week. We show analytically that the optimal strategy for early detection of emerging outbreaks is targeting individuals with the highest eigenvector centrality, a measure that considers the connectivity of a node’s neighbors, and those neighbors’ neighbors, and so on [53]. It can only be calculated with full knowledge of the network, and estimates the proportion time spent on a node during an infinitely long random walk along the edges of the network. While providing the longest lead time (between the surveillance system crossing a prevalence threshold and the rest of the population crossing that threshold), the timing is highly dependent on *R*_0_. In fact, regardless of which nodes are under surveillance, epidemiological activity becomes more synchronized and the lag time shrinks as *R*_0_ increases.

This ideal scenario is generally unrealistic. When the contact network is unknown, we cannot easily identify the most central individuals, for many measures of centrality. Even if we could monitor the most connected individuals, correct interpretation of the resulting signal requires some knowledge of *R*_0_. In general, low *R*_0_ implies a longer lag time between epidemiological events in the surveillance group and corresponding events in the general population, and a larger discrepancy between prevalence in the surveillance group and overall epidemiological activity. Several recent studies have identified epidemiologically relevant measures of centrality that can be estimated from readily obtainable school, social network, and workplace data [42, 43, 47, 48]. We hypothesize that these more tractable centrality-based sensors may exhibit a similar trade off between timeliness and robustness.

The random acquaintance strategy, which chooses random contacts of random nodes, provides a practical method for identifying individuals with higher than average centrality. The intuition is that when choosing a random *friend of a node* rather than just a *random node*, the choice is biased towards individuals with more friends. In heterogeneous networks, such as the scale-free and Montreal WiFi network considered here, random acquaintance groups provide some degree of early warning (significantly more than randomly selected nodes) and exhibit epidemic curves that reflect overall disease activity (significantly better than the most connected nodes). This is corroborated by the empirical finding that friends of random students served as better outbreak sentinels than random students during 2009 H1N1 pandemic [46]. Although the timing of the early warning and the discrepancy between the estimated prevalence and true prevalence will depend on *R*_0_, the uncertainty can potentially be quantified and incorporated into confidence intervals.

In a relatively homogeneous network, such as the Venezuelan student network, the random acquaintance strategy finds fairly average nodes and does not improve upon the random strategy with respect to the surveillance objectives. This finding is consistent with basic theory on Erdős-Réyni networks: in a random network with a Poisson degree distribution, the average degree of random acquaintances will be exactly average [57]. Therefore, if a population is sufficiently homogeneous, surveillance systems should simply target random individuals or employ other methods for identifying highly connected individuals.

We conclude that the friends-of-random strategy, while not optimal for all public health objectives, balances risk and representativeness, provides reasonably robust, accurate and early warning, and can be applied without knowledge of the underlying contact network. Volunteer-based surveillance systems, like Flu Near You, could potentially improve coverage by recruiting friends of existing members. Network analysis, in general, allows us to anticipate individual-level epidemiological risk and can thereby help us improve and strategically extend surveillance systems to enhance the early and reliable identification of outbreaks.

## Supporting Information

S1 Fig Venezuelan students network. Key features of the Venezuelan students network
(JPEG)

S2 Fig Comparison of strategies. Performance of the top-dregree (green), eigenvector centrality (red) and k-shell decomposition strategies when calculating the Peak time difference (left) and Peak ratio (right) for the three networks used: Scalefree (top), Students (middle) and Montreal (bottom).
(JPEG)
