# Supplementary figures and images for: Disease Surveillance on Complex Social Networks
Source: PLoS Comput Biol. 2016 Jul 14;12(7):e1004928. doi: 10.1371/journal.pcbi.1004928 (PMC4944951; doi:10.1371/journal.pcbi.1004928)

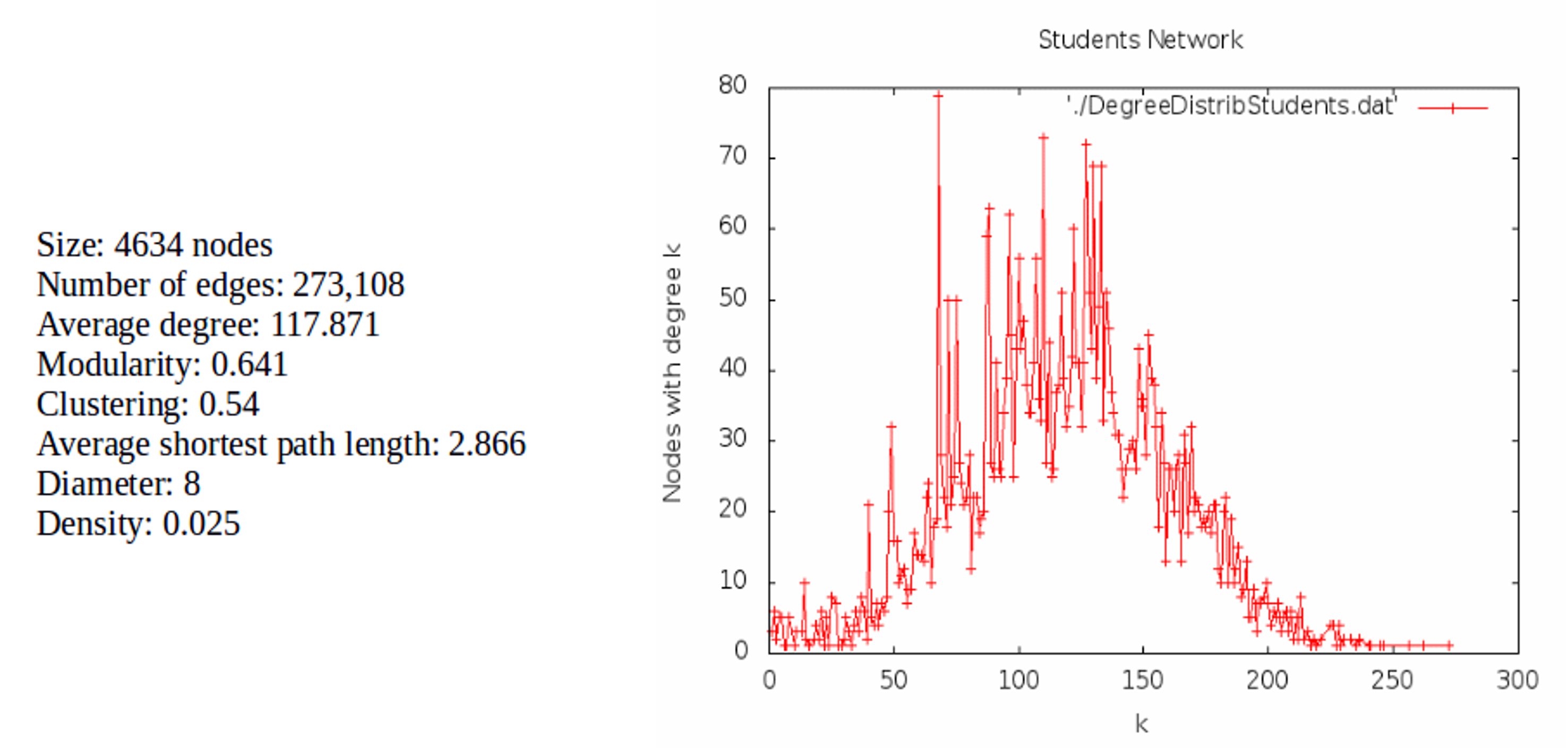

Supplement: S1 Fig — Key features of the Venezuelan students network (JPEG) [file pcbi.1004928.s001.jpeg]

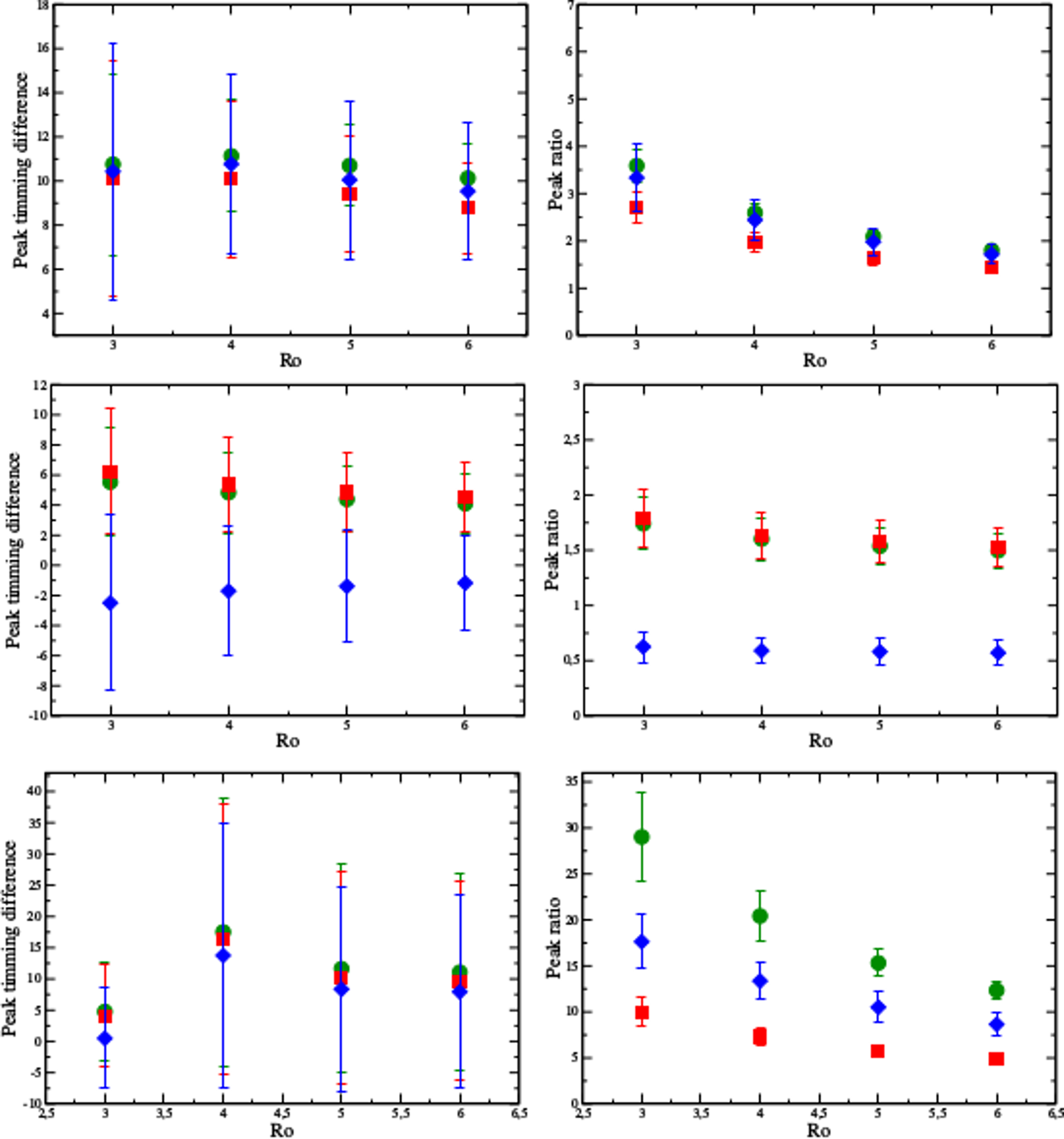

Supplement: S2 Fig — Performance of the top-dregree (green), eigenvector centrality (red) and k-shell decomposition strategies when calculating the Peak time difference (left) and Peak ratio (right) for the three networks used: Scalefree (top), Students (middle) and Montreal (bottom). (JPEG) [file pcbi.1004928.s002.jpeg]
